# ApoER2: Functional Tuning Through Splicing

**DOI:** 10.3389/fnmol.2020.00144

**Published:** 2020-07-31

**Authors:** Christina M. Gallo, Angela Ho, Uwe Beffert

**Affiliations:** ^1^Department of Biology, Boston University, Boston, MA, United States; ^2^Department of Pharmacology and Experimental Therapeutics, Boston University School of Medicine, Boston, MA, United States

**Keywords:** apoER2, LRP8, alternative splicing, cassette exon, RNA binding proteins, synaptic plasticity, apoE

## Abstract

Alternative splicing occurs in over 95% of protein-coding genes and contributes to the diversity of the human proteome. Apolipoprotein E receptor 2 (apoER2) is a critical modulator of neuronal development and synaptic plasticity in the brain and is enriched in cassette exon splicing events, in which functional exons are excluded from the final transcript. These alternative splicing events affect apoER2 function, as individual apoER2 exons tend to encode distinct protein functional domains. Although several apoER2 splice variants have been characterized, much work remains to understand how apoER2 splicing events modulate distinct apoER2 activities, including ligand binding specificity, synapse formation and plasticity. Additionally, little is known about how apoER2 splicing events are regulated. Often, alternative splicing events are regulated through the combinatorial action of RNA-binding proteins and other epigenetic mechanisms, however, the regulatory pathways corresponding to each specific exon are unknown in most cases. In this mini-review, we describe the structure of apoER2, highlight the unique functions of known isoforms, discuss what is currently known about the regulation of apoER2 splicing by RNA-binding proteins and pose new questions that will further our understanding of apoER2 splicing complexity.

## Introduction

RNA splicing is the process by which the spliceosome, a large ribonucleoprotein complex, catalyzes the excision of introns (non-coding regions) and the ligation of exons (coding regions) to form precursor-mRNAs (pre-mRNAs). After splicing and 5′- and 3′-end processing, pre-mRNAs become mature mRNAs that can be translated into functional proteins. Alternative splicing diversifies pre-mRNAs by varying which splice sites are used, resulting in events such as cassette exon skipping and intron retention. Alternative splicing generates unique transcripts from the same gene, creating isoforms with altered stability, localization, translation competency or coding sequence (Hughes, 2006; Li et al., 2007). RNA-binding proteins (RBPs) modulate alternative splicing by interacting with the spliceosome and binding to *cis* RNA elements to regulate splice site usage (Jurica and Moore, 2003; Matlin et al., 2005). The tissue specific expression and dynamic post-translational modification of RBPs (Grabowski, 1998; Grabowski and Black, 2001; Iijima et al., 2011) work together with epigenetic modifications to regulate alternative splicing patterns throughout development and in response to extracellular cues (Vallano et al., 1999; Iijima et al., 2011; Allen et al., 2017). In the brain, alternative splicing of neurotransmitter receptors and synaptic strength modulators is often regulated by activity, or neurotransmitter stimulation of synapses (Li et al., 2007). However, little is known about the regulation and function of individual alternative splicing events.

Apolipoprotein E receptor 2 (apoER2), gene name *lrp8*, is a type I transmembrane protein of the low density lipoprotein receptor (LDLR) family. ApoER2 regulates cortical lamination during development (Trommsdorff et al., 1999), interneuron precursor migration in the rostral migratory stream (Andrade et al., 2007) and learning and memory in the adult brain (Beffert et al., 2005; Chen et al., 2005). Compared to other LDLR family members, apoER2 is enriched in the brain and displays an unusually high number of alternative splicing events (Kim et al., 1996; Clatworthy et al., 1999), particularly within neurons (Zhang et al., 2014). ApoER2 alternative splicing is enriched in cassette exon splicing events, in which entire exons are included or excluded from pre-mRNAs. To date, alternative splicing of apoER2 exons has been shown to modify ligand binding properties (Brandes et al., 2001), receptor glycosylation and processing (Wasser et al., 2014) and downstream signaling (Beffert et al., 2006). Additionally, altered apoER2 alternative splicing has been implicated in sporadic Alzheimer’s disease (sAD) (Hinrich et al., 2016), the most common form of dementia. ApoER2 binds the secreted lipid carrier apolipoprotein E (apoE) (Kim et al., 1996), of which the ε4 allele is the strongest genetic risk factor for sAD (Corder et al., 1993; Farrer et al., 1997). However, how apoER2 alternative splicing becomes altered in sAD and whether altered apoER2 alternative splicing impacts its binding to apoE is unknown.

Whereas many reviews have focused on apoER2 function in brain development and adult synaptic plasticity (Reddy et al., 2011; Holtzman et al., 2012; Lane-Donovan and Herz, 2017), this mini-review will describe apoER2 structure and known function and regulation of apoER2 splice variants in the brain. We will also highlight key questions for future studies investigating apoER2 alternative splicing.

## ApoER2 Structure

**FIGURE 1:**
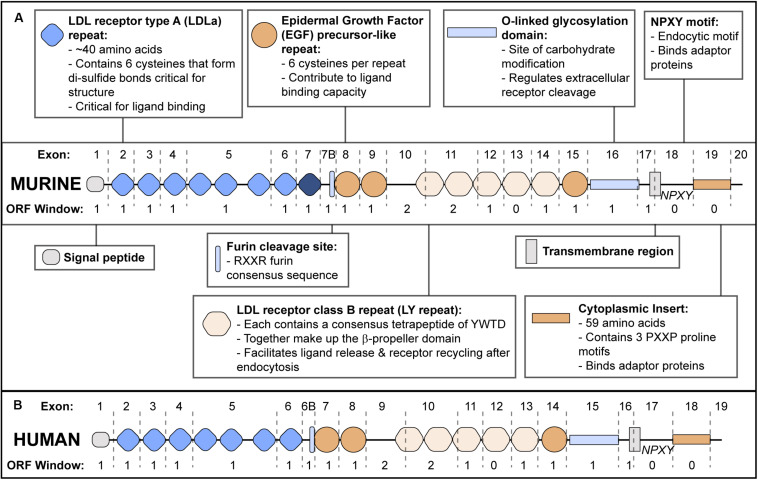
ApoER2 Exon Structure and Protein Functional Domains. **(A)** Murine apoER2 protein structure with corresponding exon boundaries (dashed lines) and protein domains functionally annotated. The eighth LDLa repeat (highlighted in dark blue) is unique to mice. Open Reading Frame (ORF) window indicates number of nucleotides at the 3′ end of each exon that require nucleotides from the next exon to encode an amino acid in the correct ORF. **(B)** Human APOER2 structure.

Apolipoprotein E receptor 2 structure (reviewed in Dlugosz and Nimpf, 2018) is modular, and individual exons tend to encode discrete functional domains (Figure 1). ApoER2 contains five functional domains characteristic of the LDLR family. The N-terminal domain contains two types of binding repeats: LDL receptor type A (LDLa) and epidermal growth factor (EGF) precursor-like repeats. While mouse apoER2 contains eight LDLa repeats, analysis of the human *LRP8* gene suggests the exon encoding the eighth LDLa repeat was lost through exon shuffling (Kim et al., 1998). Therefore in mice, exon numbering is shifted compared to humans (Brandes et al., 1997).

The second functional domain is the β-propeller domain (Rudenko et al., 2002) followed by an O-linked glycosylation domain (Kim et al., 1996; Wasser et al., 2014) and a hydrophobic transmembrane region. The fifth domain is a short cytoplasmic tail consisting of a conserved endocytic motif and a 59 amino acid insert that is not present in other LDLR family members (Kim et al., 1996; Novak et al., 1996; Brandes et al., 1997; Myant, 2010).

## APOER2 Splice Variants: Function and Regulation

Below we discuss what is known regarding function, expression and regulation of each apoER2 alternative splicing event (Table 1).

**TABLE 1:** Summary of apoER2 alternatively spliced exons, including their associated *in vitro* and *in vivo* phenotypes, spatiotemporal distribution and known splicing regulators.

Alternative Exon	Functional domain affected	*In vitro* phenotype	*In vivo* phenotype	Spatiotemporal distribution	Splicing regulator	Associated publications
Murine and Human exon 5	LDLa ligand binding repeats 4–6	Required for α_2_-macroglobulin binding (HEK-293 cells)	Unknown	**Murine**: – solely Δex5 detected (total brain RNA) **Human**: – +/Δex5 detected (total brain RNA) – solely Δex5 detected in adult frontal cortex, hippocampus and cerebellum and fetal brain (RNA)	Unknown	Kim et al., 1997; Clatworthy et al., 1999; Brandes et al., 2001
Murine exon 7	LDLa ligand binding repeat 8 (lacking in humans)	– Exclusion leads to lower affinity for β-VLDL particles (HEK-293 cells) – Exclusion confers altered affinity for reelin cleavage products (COS7 cells)	Unknown	– +/Δex7 both detected (total brain RNA) – Δex7 only at E10 (total brain RNA) – +/Δex7 at E12 and E15/16 (total brain RNA) – E15/16: cerebrum contains +/Δex7 while cerebellum and olfactory bulbs soley express +ex7 (RNA) – Ex7 cerebrum expression higher at E15-17 than P10 (protein) – Ex7 cerebellar expression increases from E17 to P10 (protein) – Cerebellum contains higher ex7 levels than cerebrum at P10 (protein) – P10 cerebellum: ex7 expression in internal granule cell layer but not external granule layer or Purkinje cells (protein) – E17 ex7 expression in cortical plate, but scarce in Marginal, Intermediate and Subventricular zones (protein)	Unknown	Brandes et al., 1997, 2001; Kim et al., 1998; Koch et al., 2002; Hibi et al., 2009
Murine exon 7B (Human exon 6B)	Furin cleavage site	– Cleaved in furin dependent manner (HEK-293 cells) – Extracellular cleavage product binds reelin – Cleavage product suppresses Dab1 phosphorylation (primary neurons IP)	Cleavage product remains undetected in brain	**Murine**: – low level inclusion of ex7B (total adult and embryonic brain RNA) – mutually exclusive with ex7 (total brain RNA) – low level inclusion of ex7B in E15-16 cerebrum but none in E15-16 cerebellum or olfactory bulb (RNA) **Human**: – low level inclusion of ex6B (total brain RNA)	Preliminary evidence for Rbfox2	Brandes et al., 1997, 2001; Koch et al., 2002; Gehman et al., 2012
Murine exon 16 (Human exon 15)	O-linked glycosylation site; extracellular cleavage site	– Required for extracellular cleavage by metalloproteases (HEK-293 cells and primary neurons) – Required for receptor sugar glycosylation (CHO cells and brain lysate) – Exclusion alters hippocampal LTP and CA1 synaptic strength in exon 19 dependent manner (hippocampal slice electrophysiology)	**Murine:** **Constitutive exclusion leads to**: – increased brain apoER2 mRNA and protein levels – increase in hippocampal dendritic spines – impaired fear acquisition (context and cued fear conditioning task) – augmented glutamate receptor expression	**Human**: – +/Δex15 both detected in adult frontal cortex, hippocampus and cerebellum and fetal brain (RNA)	Unknown	Clatworthy et al., 1999; Wasser et al., 2014
Murine exon 19 (Human exon 18)	Cytoplasmic insert	– Binds adaptor proteins JIP1, JIP2, PSD-95, APBA1, APBA2 (Y2H screen, IP) – Required for reelin-induced LTP in hippocampus (hippocampal slice electrophysiology)	**Murine**: Ex19 inclusion lower in aging and amyloid mouse model hippocampus (semi-qRT-PCR) **Constitutive exclusion confers**: – deficits in hippocampal dependent learning (fear conditioning/associative learning) – decreased number of adult corticospinal neurons – decreased thickness of adult primary motor cortex – protection from corticospinal neuronal death following brain injury **Human**: – Ex18 inclusion is lower in middle temporal region of the AD brain (semi-qRT-PCR) – Ex18 inclusion associated with global cognition (linear regression models)	**Murine**: – brain stem cells solely express Δex19 – approximately equal +/Δex19 levels found in primary neurons, total brain RNA and hippocampal RNA – ex19 confirmed at protein level (total brain lysate) **Human:** – approximately equal +/Δex18 levels in adult middle temporal region, frontal cortex, hippocampus and cerebellum and whole fetal and adult brains (RNA)	– Moderate evidence for SRSF1 – Activity regulated	Kim et al., 1998; Clatworthy et al., 1999; Stockinger et al., 2000; Gotthardt et al., 2000; Beffert et al., 2005; Beffert et al., 2006; He et al., 2007; Hinrich et al., 2016

*LDLa, Low density lipoprotein receptor class A; HEK-293, Human Embryonic Kidney-293; VLDL, Very low density lipoprotein; IP, immunoprecipitation; LTP, Long term potentiation; Y2H, Yeast-2-Hybrid; qRT-PCR, quantitative real time-polymerase chain reaction.*

### Human and Murine Exon 5 and Murine Exon 7

Exon 5 (ex5) encodes LDLa binding repeats 4–6. In the murine brain, ex5 is constitutively excluded (Brandes et al., 2001). In humans, ex5 is included approximately 50% of the time in total brain RNA (Kim et al., 1997), yet is absent in RNA from fetal brain and adult frontal cortex, hippocampus and cerebellum (Clatworthy et al., 1999), suggesting ex5 splicing is under spatiotemporal regulation. Functionally, exclusion of ex5 prevents apoER2 from binding α_2_-macroglobulin (Brandes et al., 2001), a secreted protein that inhibits proteases and sequesters ligands (Cater et al., 2019). Murine variants lacking both exons 5 and 7 have lower affinity for β-VLDL *in vitro* compared to those lacking ex5 alone (Brandes et al., 2001), which could regulate lipid trafficking.

Apolipoprotein E receptor 2 also binds secreted glycoprotein Reelin (D’Arcangelo et al., 1999), which regulates neuronal migration (Trommsdorff et al., 1999) and long-term potentiation (LTP) (Weeber et al., 2002). Reelin-apoER2 binding occurs primarily between the first apoER2 LDLa repeat encoded by exon 2 (Yasui et al., 2010) and the Reelin central fragment consisting of reelin repeats 3–6 (Jossin, 2004). This affinity can be tuned both by alternative splicing of apoER2 and proteolytic cleavage of Reelin. Exclusion of the eighth LDLa repeat, encoded by exon 7 (ex7), increases apoER2’s affinity for Reelin fragments in which Reelin repeats 7–8 are cleaved from the central fragment. The eighth LDLa repeat sterically hinders binding of the Reelin central fragment, thereby modulating apoER2-Reelin affinity and subsequent signaling (Hibi et al., 2009).

Interestingly, murine ex7 expression overlaps with neuronal migration (Table 1), suggesting the eighth LDLa repeat can modulate network formation or synaptic plasticity (Hibi et al., 2009). However, this possibility has yet to be tested. As the eighth LDLa repeat is not present in human APOER2 (Kim et al., 1998), how this functionally translates to humans is unclear. The regulatory mechanisms controlling ex5 and ex7 splicing are also unknown.

### Human Exon 6B (Murine Exon 7B)

Human exon 6B encodes thirteen amino acids in the APOER2 extracellular domain including the furin consensus sequence RXXR. In the murine brain, exon 7B (ex7B) inclusion is only observed when ex7 is excluded (Brandes et al., 1997, 2001). *In vitro*, murine isoforms containing ex7B are proteolytically cleaved in a furin-dependent manner releasing a soluble extracellular product. This furin-cleaved product acts as a dominant-negative inhibitor of full-length apoER2 by binding and sequestering Reelin, therefore interfering with Reelin-apoER2 signaling. Although the soluble fragment has yet to be identified *in vivo*, it may be present at low concentrations and contribute to Reelin pathway modulation. Ex7B also appears to be developmentally regulated, as it is detectable in the cortex but not the olfactory bulb or cerebellum in murine E15-16 brains (Koch et al., 2002).

Splicing factor Rbfox2 has been identified as a possible regulator of murine ex7B splicing. In *Rbfox2*^–/–^ mice, brain apoER2 ex7B inclusion is increased compared to wild-type mice, suggesting Rbfox2 may serve as a repressor of ex7B inclusion. Five potential Rbfox2 binding sites near ex7B were identified, of which three are conserved across species (Gehman et al., 2012). To confirm Rbfox2 directly modulates ex7B inclusion, binding of Rbfox2 to apoER2 putative binding sites still needs to be demonstrated.

### Human Exon 18 (Murine Exon 19)

Human exon 18 (ex18) encodes the APOER2 cytoplasmic insert. In adult mice, exon 19 (ex19) is essential for Reelin-induced enhancement of hippocampal LTP (Beffert et al., 2005). The encoded cytoplasmic insert binds postsynaptic density protein-95 (PSD-95) (Gotthardt et al., 2000), which recruits Src family kinases to phosphorylate NMDA receptor subunits. This increases calcium conductance, facilitating LTP (Beffert et al., 2005; Chen et al., 2005). Accordingly, mice constitutively lacking ex19 (Δex19) demonstrate deficits in hippocampal-dependent learning. As Δex19 mice show no neuroanatomical changes, this learning deficit is likely due to loss of the functional apoER2-NMDA receptor complex, not altered neuronal migration (Beffert et al., 2005).

Murine ex19 alternative splicing is also involved in neural degeneration and aging, as adult Δex19 mice have fewer corticospinal neurons and a thinner primary motor cortex. Interestingly, in response to brain injury, Δex19 mice are protected from neuronal death compared to wild-type and constitutively expressing ex19 (+ex19) mice. This effect is likely due to ex19-dependent binding of JNK-interaction proteins 1 and 2 (JIP1, JIP2) (Gotthardt et al., 2000; Stockinger et al., 2000), which activate Jun N-terminal kinase (JNK) and cellular death signaling (Beffert et al., 2006). Ex19 also interacts with neuronal adaptor proteins X11α and X11β, officially APBA1 and 2 (He et al., 2007). ApoER2-APBA1/2 binding facilitates formation of a protein complex containing BACE1 and amyloid precursor protein (APP). ApoE-apoER2 binding promotes APBA1/2-dependent endocytosis of APP and BACE1 leading to Aβ production (He et al., 2007), a neuropathological hallmark of AD. However, whether the apoER2-APBA1/2 interaction affects Aβ production *in vivo* remains to be determined. Interestingly, Reelin decreases apoER2-APBA1/2 interaction in neurons (Minami et al., 2010), suggesting that Reelin and apoE binding to apoER2 modulates intracellular adaptor protein interaction and functions.

In humans, ex18 inclusion is positively correlated with global cognition. AD patients demonstrate lower apoER2 ex18 inclusion in the middle temporal cortex compared to non-cognitively impaired patients. Murine ex19 inclusion is also decreased in the hippocampus of AD mouse model TgCRND8 (APP carrying Swedish and Indiana mutations) and increasing ex19 inclusion with antisense oligonucleotides (ASOs) can partially rescue hippocampal-dependent spatial learning (Hinrich et al., 2016). It is therefore reasonable to hypothesize that apoER2 alternative splicing manipulation could be therapeutically useful for AD, especially considering the success of other ASOs in neurodegenerative diseases (Wurster and Ludolph, 2018).

Due to apoER2’s role in memory, understanding ex18 splicing regulation is critical. *Lrp8* contains two putative binding sites in the flanking 3′-intron of ex18 for serine/arginine-rich splicing factor 1 (SRSF1) that are conserved across humans and mice. Knockdown of SRSF1 or application of an ASO targeting SRSF1 binding sites increases ex18 inclusion, suggesting SRSF1 represses ex18 inclusion (Hinrich et al., 2016). However, it is unclear whether the ASO-induced increase in ex18 inclusion is dependent on SRSF1. Periods of activity also regulate ex19 in the murine brain, with higher inclusion during periods of feeding and lower inclusion during less active periods (Beffert et al., 2005). Exactly how regulation occurs during periods of activity and whether it involves SRSF1 is unknown.

### Human Exon 15 (Murine Exon 16)

Human exon 15 encodes the O-linked sugar domain of APOER2, the site of O- and N-linked glycosylation. This region contains an extracellular cleavage site for matrix metalloproteinases (MMPs), like ADAM10, as murine exon 16 (ex16) exclusion prevents apoER2 cleavage (Wasser et al., 2014). Reelin-apoER2 binding stimulates MMP cleavage of the receptor followed by a second intramembranous cleavage by γ-secretase. This releases the intracellular domain (ICD) which translocates to the nucleus and activates an enhancer profile necessary for transcription of learning and memory genes (Telese et al., 2015). As ex16 encodes the MMP cleavage site, ex16 inclusion regulates the apoER2 proteolytic pathway and likely ICD translocation. However, a conclusive link between ex16 inclusion and ICD translocation has yet to be established. Of note, the ICD contains the cytoplasmic insert encoded by ex19, which could affect enhancer activation.

Constitutive ex16 exclusion (Δex16) confers higher apoER2 levels in the murine brain, both at the protein and mRNA level. Tandem deletion of ex19 (Δex16/Δex19) in mice exacerbates this effect (Wasser et al., 2014), suggesting that apoER2 splicing impacts total apoER2 expression. Precise control of apoER2 expression is critical, as overexpression and knockout lead to increases and decreases, respectively, in dendritic spines, suggesting that apoER2 affects synapse formation (Dumanis et al., 2011). Synaptic apoER2 levels are also post-translationally regulated by E3 ubiquitin ligase Inducible Degrader of the LDL receptor (IDOL). Neuronal activity downregulates IDOL, allowing apoER2 levels to increase and initiate cytoskeletal remodeling and LTP through induction of the GTPase Rac1. ApoER2’s binding of JIP1/2 may modulate Rac1 activation as JIP1/2 also bind Tiam1, the guanine nucleotide exchange factor that ties NMDAR to Rac1 activation (Gao et al., 2017). While IDOL ubiquitinates apoER2 within constitutive murine exon 18, JIP1/2 binds the cytoplasmic insert (Stockinger et al., 2000), suggesting that alternative splicing may influence this pathway.

Due to elevated apoER2 levels, Δex16 mice display increased CA1 hippocampal spine numbers yet weaker synapses. Tandem exclusion of ex19 exacerbates both phenotypes. While Δex16/Δex19 mice show the larger increase in apoER2 expression and spine numbers, it is Δex16 mice constitutively expressing ex19 (Δex16/+ex19) that show augmented LTP compared to wild-type. As this difference can be rescued by reducing apoER2 expression (Wasser et al., 2014), it is curious that Δex16/Δex19 mice do not demonstrate a similar or stronger LTP response. This is also intriguing given +ex19 mice alone show no LTP or synaptic strength alterations (Beffert et al., 2005), suggesting for the first time *in vivo* that tandem apoER2 splicing events can have unique synaptic effects. To parse out which effects depend on ex16 or ex19, a study that compares mice with individual and double exon manipulations would be beneficial.

Exclusion of ex16 impairs murine fear acquisition, with the effect again modulated by ex19 inclusion and rescued by reducing apoER2 levels (Wasser et al., 2014). Δex16/Δex19 mice demonstrate increased glutamate receptor subunit expression of AMPARs and NMDARs as well. This is likely due to the increased apoER2 protein observed, as apoER2 binds NMDA subunits extracellularly (Hoe et al., 2006) and increases dendritic spine numbers (Wasser et al., 2014). The mechanisms regulating ex16 splicing, and if they co-regulate ex19 splicing, remain to be determined.

### Additional APOER2 Splicing Events

Several additional *APOER2* exons are alternatively spliced in the human brain, such as exon 6 (LDLa repeat 7) and exons 7 and 8 (EGF repeats) (Brandes et al., 1997; Kim et al., 1997; Clatworthy et al., 1999; Myant, 2010). However, the functional properties of these mRNAs are unknown. In humans, there is a 73-nucleotide insertion sometimes included between exons 7 and 8 that introduces a frame-shift and early stop codon and is observed in the fetal but not adult brain (Brandes et al., 1997; Clatworthy et al., 1999). These events are also observed in combination, such as exclusion of both human exons 5 and 6 (Kim et al., 1996) and exclusion of both human exons 5 and 8 (Clatworthy et al., 1999). Despite the identification of these splicing events over 20 years ago, their effect on apoER2 function remains to be determined.

## APOER2 Transcript Stability

RNA-binding proteins also affect apoER2 transcript stability. The RBP SRSF11 is downregulated in aged murine and primate prefrontal cortex and induces age-associated decline, partly through modulating apoER2 expression. SRSF11 binds directly to the 3′-UTR of *apoER2*, enhancing transcript stability. Interestingly, SRSF11 binding also regulates *apoE* mRNA stability. ApoE and apoER2 levels modulate JNK pathway activation and age-related neuronal loss, making SRSF11 a key RBP in the aging brain (Raihan et al., 2019). It remains to be seen whether apoER2 alternative splicing is involved in the SRSF11-apoER2-JNK pathway, although it seems likely as apoER2 ex19 is required for apoER2-JIP1/2 interaction (Beffert et al., 2006). The mechanism underlying decreased SRSF11 expression in aging is also unclear. The roles of SRSF11, SRSF1 and Rbfox2 highlight how multiple RBPs must be regulated to exert a concerted effort on apoER2 splicing and expression levels, which are also affected by apoER2 isoforms themselves.

## Conclusion and Future Perspectives

Overall, it is clear that alternative splicing modulates apoER2 synaptic function in the adult and aging brain. However, the function of apoER2 alternative splicing in brain development is less defined. ApoER2 ex7B, for example, is well placed to modulate neuronal migration due to its expression profile (Table 1; Koch et al., 2002) and the importance of implicated regulator Rbfox2 in cerebellar development (Gehman et al., 2012). Investigating apoER2 alternative splicing during development will require precise manipulation of exon inclusion through techniques such as splice site switching ASOs, RNA-guided dCas9 (Konermann et al., 2018) and targeted mouse genetics. Our current understanding of apoER2 alternative splicing is limited by the incomplete spatiotemporal characterization of each isoform. Future studies evaluating apoER2 variants must be quantitative, robust and include multiple time points and brain regions.

Advances in single-cell technology and sequencing have highlighted the importance of alternative splicing in defining neuronal subtypes. Recent data from ribosome-engaged transcript profiling in genetically defined murine forebrain neurons have revealed that apoER2 alternative splicing may be partially subtype specific. Additional apoER2 alternative splicing events were identified, including alternative 5′- and 3′-splice acceptor sites, intron retention and alternative transcriptional start sites, suggesting multiple levels of alternative splicing complexity regulate apoER2 (Furlanis et al., 2019). Traditional approaches like qRT-PCR can be coupled with electrophysiology to match cell-type specific firing properties with possible cell-type specific alternative splicing events. Furthermore, recent technological advances like BaseScope ultra-sensitive *in situ* hybridization (Erben et al., 2018) enable isoform localization *in vivo*. This level of cell-type specificity must become a pillar in the splicing field to truly grasp alternative splicing intricacy.

Establishing cell-type alternative splicing patterns will also help identify RBPs that regulate apoER2 splicing events in those subtypes. Numerous technologies are available to identify apoER2 RNA-RBP interactions, often incorporating unbiased mass spectrometry (Spiniello et al., 2019). Beyond the effect of RBPs on alternative splicing of individual apoER2 exons, regulatory pathways and epigenetic factors modulating RBPs are another layer of complexity that must be understood. Furthermore, human alternative splicing regulation must be investigated dynamically, likely in stem cell-derived induced-neurons (Zhang et al., 2013).

Moving forward, the role of synaptic modulators like apoER2 will only be understood in physiological and disease states once distribution, function and regulation of all their isoforms is defined. From there, perhaps targeted manipulation of the splicing code can provide future therapeutic interventions. ASOs have shown therapeutic efficacy in correcting pathogenic splicing deficits, such as Nusinersen 1 in spinal muscular atrophy (Wurster and Ludolph, 2018). Interfering with the spliceosome or splicing factors themselves using small molecules offers another potential intervention point, although less specific. This strategy is being utilized in cancer research and has helped define splicing regulatory pathways (Effenberger et al., 2017). Overall, defining the splicing code for both apoER2 and other synaptic proteins is the next frontier for understanding how the brain develops, matures and ages.

## Author Contributions

CG drafted the manuscript. All authors wrote and revised the manuscript.

## Conflict of Interest

The authors declare that the research was conducted in the absence of any commercial or financial relationships that could be construed as a potential conflict of interest.
